# Grouping of Experimental Conditions as an Approach to Evaluate Effects of Extremely Low-Frequency Magnetic Fields on Oxidative Response in *in vitro* Studies

**DOI:** 10.3389/fpubh.2014.00132

**Published:** 2014-09-02

**Authors:** Mats-Olof Mattsson, Myrtill Simkó

**Affiliations:** ^1^Environmental Resources and Technologies, Department Health and Environment, AIT Austrian Institute of Technology, Tulln, Austria

**Keywords:** mammalian cells, immune-relevant cells, flux density, exposure duration, ROS

## Abstract

A large body of literature deals with biological effects of extremely low-frequency magnetic fields (ELF MFs) studied *in vitro*. Despite the multitude of studies, no coherent picture has evolved regarding the plausibility of effects at low-flux densities or regarding the interaction mechanisms. Here, we propose that ELF MF exposure *in vitro* causes changes in oxidative status as an early response. We tested this hypothesis by scrutinizing the literature and applying a grouping approach for analyzing relevant biological properties and exposure conditions. A total of 41 scientific original publications were analyzed for this purpose. The conclusion from the work is that ELF MF (modulated or unmodulated) consistently can influence the oxidative status, at or above 1 mT, in a broad range of cell types and independent of exposure duration. A response at lower flux densities is seen in certain studies, although not consistently. Further studies with stringent protocols for sham exposure, blinding, and statistical analysis as well as appropriate positive controls are needed to establish if true dose-relationships for effects on oxidative status exist.

## Introduction

Extremely low-frequency magnetic fields (ELF MFs; 1–300 Hz) are widely present in the modern society. Such MFs originate primarily from distribution and usage of electricity and are typically found at higher magnetic flux densities in the vicinity of power lines and devices using strong electric currents. For decades, epidemiological as well as experimental studies have addressed possible health effects of exposure to these and also to higher frequency (electro)MF. Regarding chronic health effects, the International Agency for Research on Cancer (1) has classified low-frequency MF as a “possible carcinogen” (IARC class 2B). This classification is based on indirect evidence, i.e., epidemiological findings of increased risk for childhood leukemia in domestic settings where the MF-level is higher than the commonly found levels (daily averages exceeding 0.3–0.4 μT) (2, 3). However, there is no supporting evidence for this classification from animal experiments. Furthermore, there are no mechanistic data that can provide an explanation for any effect on biological structures at the flux density levels that have been identified in the epidemiological studies.

It has been established that acute effects on excitable tissues (nerve and muscle) can occur at magnetic flux densities that are much higher than the ones associated with an increased risk for childhood leukemia (at millitesla levels compared to tenths of microtesla). Current exposure guidelines, such as those published by the international commission on non-ionizing protection [e.g., Ref. (4)], are set to protect against such established effects. Although the epidemiological findings of the association between MF exposure and childhood leukemia are indicating that chronic average exposures above 0.3–0.4 μT are having an effect on disease development, there is no evidence for any causal relationships, and no other long-term effects have been established.

The scientific literature contains many experimental studies reporting various biological effects of exposure to ELF MF [see, e.g., comprehensive overviews in the opinions of SCENIHR (5, 6)]. The relevance for any disease outcome from these studies is unclear. Although these studies have been performed for decades, they have not provided with any convincing evidence for mechanistic explanations of any biological effect at low-flux density levels (at or below ICNIRP reference levels) and have not provided with consistent findings that are supporting epidemiological data.

The reported effects from *in vitro* studies include virtually all sorts of end points. This includes effects on DNA structure, gene expression, cell growth and survival, cellular metabolism, motility, protein functions, etc. It has to be noted, however, that despite the considerable number of studies with observed effects, studies not finding any effects are also very common. There is thus considerable inconsistency in the published literature. Unlike the situation in, e.g., chemical toxicology, in the case of observed effects, many studies have not addressed the issue of dose–response relationships. There is also a clear lack of systematic approaches to other important characteristics such as exposure duration.

Here, we focus on the status of oxidative responses, including free radical release, after MF exposure as an example of a biologically relevant endpoint. Free radicals, or as the term we use in the present paper, reactive oxygen species (ROS), are atoms or molecules that contain one or more unpaired electrons, which makes free radicals highly reactive, striving to form pairs to counteract the labile unpaired condition. Free radicals gain electrons from any available donor or donate an electron to a suitable acceptor, which in turn becomes modified into a secondary free radical. This chain reaction can cause biological damage leading to macromolecule damage such as DNA modifications or protein oxidation.

During aerobic conditions, free radicals are produced during and through normal metabolic processes. Key sources include electron transfer in the plasma membrane and cell respiration in the mitochondrial membrane. The production can proceed enzymatically or non-enzymatically. Shigenaga et al. (7) suggested that the mitochondria are the main source of the oxidative damage because free radicals such as superoxide can escape from the electron transport chain. About 3–10% of the oxygen turned over there is not fully processed, i.e., reduced. ROS from the mitochondria can enter the cytosol and react with other substances and thereby form new radicals. This triggers a chain reaction in which electrons change their owners, which can lead to DNA modification or enzyme disruption.

In order to counteract intracellular damage by free radicals, cells have antioxidant systems. These transform free electrons into a non-reactive form by proteins or enzymes. Antioxidants regulate oxidative reactions by inhibiting, delaying, or hampering the oxidation of the molecules (8). The intracellular enzymes that function as antioxidants are the backbone of this cellular defense system (9, 10). The key antioxidant enzymes are catalase (CAT), superoxide dismutase (SOD), and various peroxidases. In addition, non-enzymatic antioxidants can also neutralize radicals (e.g., vitamin C, E or A/β-carotene, glutathione, and melatonin) (8).

Free radicals have a series of important functions, such as serving in the immune defense. Leukocytes and macrophages execute their bactericidal effects by the release of ROS as a cellular defense mechanism against entering pathogens, thus, killing bacteria, viruses, and degenerated cells. At low concentrations, ROS can act as second messengers and activate signaling cascades, which in turn can lead to physiological responses such as gene expression, cell proliferation, and apoptosis [for reviews see Ref. (11, 12)]. However, immune-relevant cells use the reactive potential of ROS also to fulfill important physiological functions such as regulating the vascular tone and those cell functions controlled by oxygen concentration. Oxidative stress is the result of an imbalance between the intracellular ROS production and the cellular defense mechanisms. The balance between oxidants and antioxidants, the redox homeostasis, can be disrupted by an increase in free radicals or a reduction of anti-oxidative substances. Depending on the duration and strength of the imbalance, the redox regulation of the cell fulfills a compensatory function. When a constant production of free radicals is triggered by oxidative stress, the redox homeostasis becomes unbalanced and the cellular mechanisms are no longer capable of establishing the normal levels. This can not only persistently change signal transduction but also lead to changes on gene and protein levels and thus further promote oxidative conditions or processes. This includes virtually all complex molecules that can gain a single electron (DNA, proteins, lipids, and carbohydrates).

Despite that numerous investigations have shown the presence of a multitude of biological effects of ELF MF exposure, the first point of interaction between MF and cells, and the underlying molecular mechanisms, are still not clear. However, the release of ROS or other oxidative processes are often connected to the investigated effects. Therefore, we assume that oxidative processes triggered by MF play a key role within the effectiveness of MF (13, 14). Here, we focus on *in vitro* studies investigating the oxidative processes after exposure to low-frequency MF. Our hypothesis is that MF exposure consistently can trigger oxidative responses in cultured mammalian cells. Moreover, we apply a grouping approach to analyze relevant biological or exposure conditions for MF triggered oxidative processes.

We have asked a set of questions regarding (exposure) conditions and biological responses:

- Are responses related to the cell type used in the study?
- Are effects related to the magnetic flux density?
- Are the effects related to modulation of the MF signal?
- Are the responses related to exposure duration?

In addition, we have also evaluated certain quality criteria in the studies, such as whether or not true sham exposure conditions have been employed; if blinded protocols have been used; and if the appropriate positive controls have been included in the studies. Finally, we investigate the “effect size” of the response in the form of ROS production.

## Approach and Outcomes

We have identified (PubMed^1^ and EMF-Portal^2^) 41 published papers in the English language dealing with oxidative processes after ELF MF exposure of mammalian cells *in vitro*. Many of these studies have already been extensively reviewed (5, 6, 13–15). Therefore, our goal is to evaluate these studies by identifying and using a “grouping” tool to classify relevant conditions, such as cell type, and/or exposure conditions, for cellular response after MF exposure and not to perform a comprehensive review. We are aware of the limited number of relevant studies and also about the inadequate quality of some of the investigations. Consequently, some additional studies have not been included in this article since those studies have not matched our inclusion criteria, which include the possibility to follow the exposure and experimental approach. We were especially excluding studies where exposure or other experimental conditions were inappropriate and/or unsatisfactory described. The level of criteria is admittedly low, but was accepted on practical grounds, otherwise only few publications would have been taken into account.

First of all, we have considered “positive” and “negative” findings among the publications. A “positive” study refers to a study where an effect of MF is shown, with valid methods described in enough detail to constitute evidence supporting the study hypothesis. If a well-conducted and appropriately reported study shows no clear effect despite proper methods and statistical power, its results provide evidence against the study hypothesis (but support the null hypothesis), and the study is considered “negative” (Table 1).

**Table 1:** **Summary of identified relevant studies**.

Cells				Exposure conditions	Oxidative response^a^	Reference
Human peripheral blood mononuclear cells	Mononuclear cells	Primary	Immune relevant	20–5000 Hz 5 μT 30 min	No	(16)
Human peripheral blood mononuclear cells	Mononuclear cells	Primary	Immune relevant	50 or 60 Hz 2, 20, 100, 500 μT 6 h	No	(17)
MCF10A human breast epithelial cells	Breast epithelial cells	Cell line	Other cell line	60 Hz 1 mT 4 h	No	(18)
Murine L929 fibroblasts	Fibroblasts	Cell line	Other cell line	50 Hz 100 or 300 μT 1 or 24 h	No	(19)
Rat-cortical neurons	Neurons	Primary	Other primary cells	50 Hz 0.1 and 1.0 mT 7 days	No	(20)
Rabbit red blood cells	Red blood cells	Primary	Other primary cells	50 Hz 0.2, 0.5 mT 45, 90 min	No	(21)
THP-1 cells (human monocytic leukemia cell line)	Leukemia	Cell line	Immune relevant	50 Hz 1 mT 4 h	Yes	(22)
K562 cells	Myelogenous leukemia	Cell line	Immune relevant	50 Hz 1 mT 3 h	Yes	(23)
K562 cells	Myelogenous leukemia	Cell line	Immune relevant	50 Hz 5 mT 1 h	Yes	(24)
Murine osteoblasts (7F2) co-cultured with RAW 264.7 macrophages	Osteoblasts, macrophages	Cell line	Immune relevant	75 Hz, PEMF 1.5 mT 9 h	Yes	(25)
Human mono Mac 6 cells	Monocytic leukemia	Cell line	Immune relevant	50 Hz	Yes	(26)
Human umbilical cord blood-derived monocytes	Monocytes	Primary		1 mT 45 min		
K562 cells	Myelogenous leukemia	Cell line	Immune relevant	50 Hz 0.025–0.10 mT 1 h	Yes	(27)
THP-1	Monocytic cells	Cell line	Immune relevant	50 Hz 1 mT 6, 24 h	Yes	(28)
Primary mouse macrophages	Macrophages	Primary	Immune relevant	50 Hz 0.05–1.0 mT 45 min–48 h	Yes	(29)
Mouse macrophages from isolated bone marrow precursor cells	Macrophages	Primary	Immune relevant	50 Hz 1 mT 5 min–24 h	Yes	(30)
Human blood platelets from voluntary donors	Platelets	Primary	Immune relevant	50 Hz 10 mT 15 min	Yes	(31)
Human peripheral blood mononuclear cell	Mononuclear cells	Primary	Immune relevant	50 Hz 1, 3, 10, 30 mT 3 days	Yes	(32)
Human peripheral blood mononuclear cell from Cohn’s disease patients	Mononuclear cells	Primary	Immune relevant	PEMF 50 Hz 45 mT 3 h/day × 3 h/day	Yes	(33)
Human umbilical cord blood-derived monocytes	Monocytes	Primary	Immune relevant	50 Hz 1 mT 5–45 min	Yes	(34)
Peritoneal neutrophils isolated from male SD rats	Neutrophils	Primary	Immune relevant	60 Hz 0.1 and 2.0 mT up to 1000 s	Yes	(35)
Human peripheral blood neutrophils	Neutrophils	Primary	Immune relevant	180–195 Hz PEMF 10, 40, 60 μT n.n.	Yes	(36)
Human monocyte	Monocytes	Primary	Immune relevant	50 Hz 1 mT Overnight	Yes	(37)
Mouse bone marrow-derived promonocytes and macrophages	Macrophages	Primary	Immune relevant	50 Hz 1 mT 45 min or 24 h	Yes	(38)
Peritoneal neutrophils from SD rats (primed)	Neutrophils	Primary	Immune relevant	60 Hz 0.1 mT up to 600 s	Yes	(39)
Murine bone marrow-derived macrophages	Macrophages	Primary	Immune relevant	50 Hz 1.0 mT 45 min	Yes	(40)
Human peripheral blood neutrophils	Neutrophils	Primary	Immune relevant	75 Hz PEMF 2.5 mT 60 min	Yes	(41)
Lymphocytes from male albino Wistar rats	Lymphocytes	Primary	Immune relevant	50 Hz 20, 40, 200 μT 1 h	Yes	(42)
AT478 murine squamous cell carcinoma cells	Squamous cell carcinoma	Cell line	Other cell line	50 Hz 1 mT 16 min	Yes	(43)
Human colon adenocarcinoma cells (Caco 2)	Colon adenocarcinoma	Cell line	Other cell line	50 Hz 1 mT 24, 48, 72 h	Yes	(44)
Human SH-SY5Y neuroblastoma cells	Neuroblastoma	Cell line	Other cell line	50 Hz 1 mT 1 h	Yes	(45)
Human prostate cancer cell lines DU145, PC3, LNCaP	Prostate cell lines	Cell line	Other cell line	60 Hz 1 mT 3, 6, 24, 48, 72 h	Yes	(46)
Human SH-SY5Y neuroblastoma cells	Neuroblastoma cells	Cell line	Other cell line	50 Hz 0.1 mT 24 h	Yes	(47)
PC12 cells	Neuronal pheochromocytoma	Cell line	Other cell line	50 Hz 0.1 or 1.0 mT 30 min 1–7 days	Yes	(48)
C2C12 cells (muscle cells)	Myoblast	Cell line	Other cell line	50 Hz 0.1 and 1.0 mT 5 and 30 min	Yes	(49)
Human keratinocyte cells HaCaT	Keratinocytes	Cell line	Other cell line	50 Hz 1 mT 3, 18, or 48 h	Yes	(50)
AT478 murine squamous cell carcinoma line	Squamous cell carcinoma	Cell line	Other cell line	400 Hz 0.11 mT 16 min	Yes	(51)
Human keratinocyte cell line HaCaT	Keratinocytes	Cell line	Other cell line	50 Hz 1 mT 4, 12, 72 h	Yes	(52)
Rat-1 fibroblasts	Fibroblasts	Cell line	Other cell line	50 Hz 1.0 mT 3 or 24 h	Yes	(53)
AT478 murine squamous cell carcinoma line	Squamous cell carcinoma	Cell line	Other cell line	3 Hz−3 kHz 0.11 mT 16 min	Yes	(54)
3T3-L1 preadipocytes	Preadipocytes	Cell line	Other cell line	180–195 Hz PEMF 0.12 mT 36 min	Yes	(55)
Human bone marrow mesenchymal stem cells (hBM–MSCs)	Mesenchymal stem cells	Primary	Other primary cells	50 Hz 1 mT 90 min	Yes	(56)

*^a^Yes, positive finding; no, negative finding*.

Here, we consider *oxidative response* as the first “grouping” of biological endpoints. All positive findings (independent of whether the response is an increase or a decrease compared to the control situation) were taken into account after exposure to MF, where any kind of oxidative response related data has been detected. These include changes not only in ROS production, in expression/activity of antioxidants, intermediate-release such as inflammation related cytokines, nitrogen oxides, but also in relevant protein expressions and/or phosphorylation.

Further on, the number of different cell types was identified and compiled into the following groups: immune-relevant primary cells, immune-relevant cell lines, other primary cells, and other cell lines (Table 1; where a summary of identified relevant studies is presented). Within the two cell line groups, cancer or non-cancer cells were sub-grouped. However, data are not shown since we could not identify any trend.

The main and most relevant exposure conditions were identified as the frequency of the applied field, frequency modulations including pulsed-electromagnetic fields (PEMF), the flux density, and the exposure duration. These conditions were sub-grouped into larger units to identify relevant conditions for positive or negative findings.

**Figure 1:**
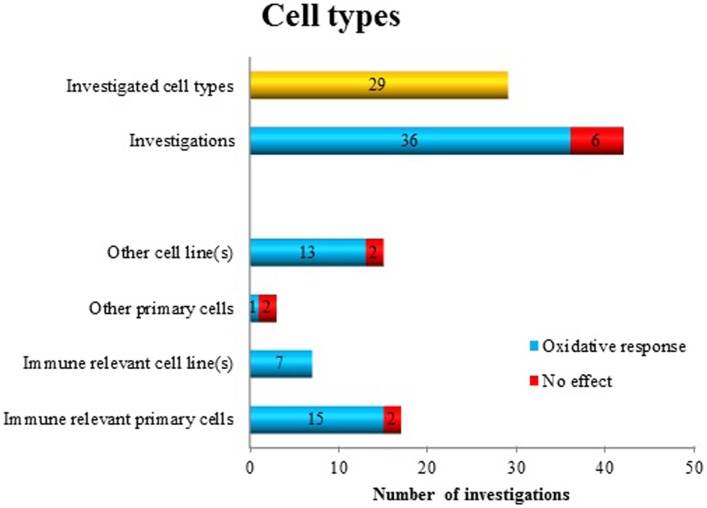
**Oxidative response as a positive or negative finding after exposure to ELF MF in different cell types**.

Within these 41 publications, 42 different “datasets” have been identified utilizing 29 different *cell types* (Figure 1). The majority of the studies, namely 36, reported positive findings regarding oxidative responses after MF exposure (corresponding to a positive finding ratio of 0.86). Some investigators used different cell types for comparative analysis. As shown in Figure 1, there is no clear trend indicating a cell type dependent MF effect. However, by pooling immune-relevant cells (independent if primary cells or cell line), 22 positive findings were detected (out of 24). Using non-immune-relevant cells or cell lines, out of 18 investigations, 14 showed positive findings.

**Figure 2:**
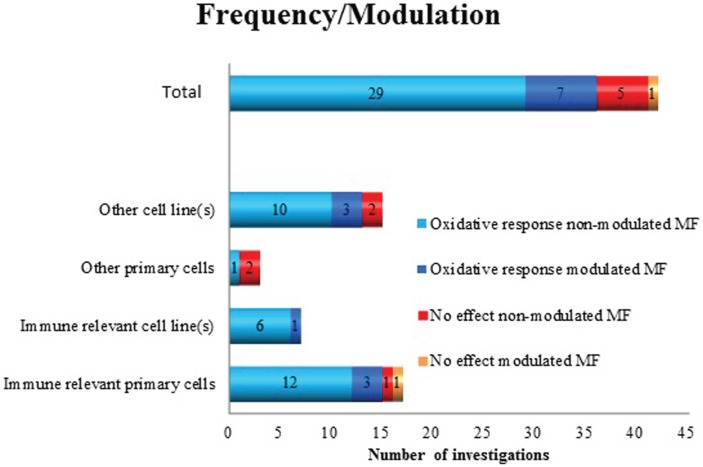
**Oxidative response as a positive or negative finding after exposure to non-modulated or modulated ELF MF in different cell types**.

By analyzing the employed *frequencies*, we considered non-modulated sine wave ELF MF as a “group” (almost all studies were at 50/60 Hz) and modulated MF as a second group where PEMF or other frequency modulations or waveforms were used. Only few studies investigated the effect of modulated MF. Of these, seven studies showed positive findings and one no effect. The majority of the studies, namely 34, employed non-modulated MF. Among those, 29 investigations detected oxidative response, and 5 detected no effects (Figure 2). Because of the imbalance between the number of studies of “modulated” and “non-modulated” (34 vs. 8) frequencies, it is not possible to detect an association or trend whether the frequency parameter is relevant for a biological response.

**Figure 3:**
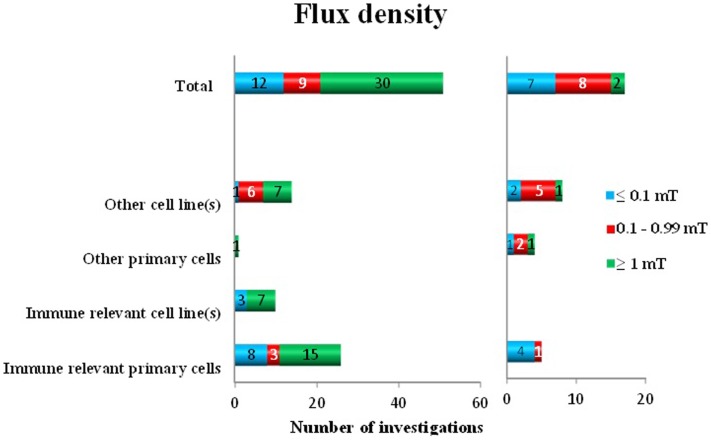
**Oxidative response as a positive or negative finding after exposure to different flux densities of ELF MF in different cell types**.

To analyze the *flux density* dependency of the performed studies, we grouped the employed field strength as follows: ≤0.1, 0.1–0.99, and ≥1 mT. Some investigators used several different flux densities. Out of 68 investigations, 51 detected positive findings: 30 used ≥1 mT, 9 used 0.1–0.99 mT, and at 12 times ≤0.1 mT was employed. No effects were detected in a total of 17 investigations (2 in ≥1 mT, 8 in 0.1–0.99 mT, and 7 in the group of ≤0.1 mT). It seems that below 0.1 mT and around 1 mT oxidative response appears in the majority of the used immune-relevant primary cells (23) (Figure 3). Based on this data, it is plausible to suggest that ≥1 mT induces oxidative responses (30) with a ratio of 0.94. At the other flux density levels, this distinct picture does not appear. The ratio for positive findings is in the group of <0.1 mT 0.63 and in the group of 0.1–0.99 mT 0.53.

**Figure 4:**
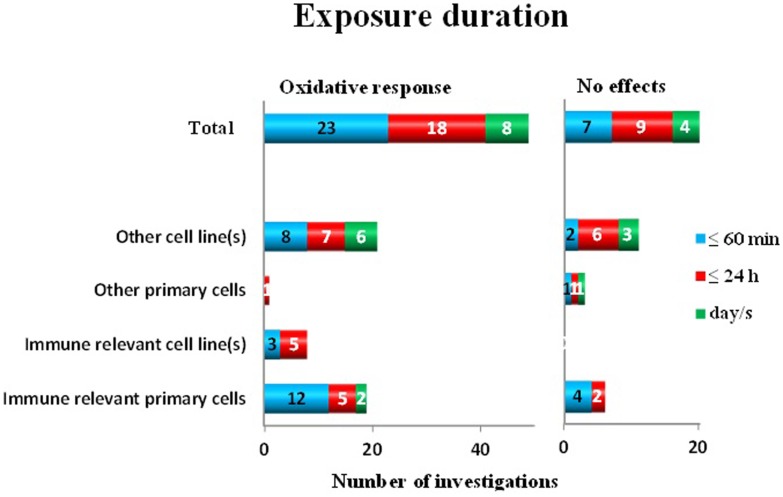
**Oxidative response as a positive or negative finding after different exposure durations to ELF MF in different cell types**.

It has been discussed that the *exposure duration* is another important condition for bioeffects. To identify if this is the case, we introduced three groups of exposure durations: ≤60 min, >1 h up to 24 h, and more than 1 day. Out of 69 investigations, oxidative response was shown in 49 studies. It seems that the exposure duration is not relevant for the biological response (Figure 4). Short exposure (up to 60 min) resulted in 23 papers in an effect, whereas no effects were reported in 7 papers (a ratio of 0.77). MF exposure up to 24 h resulted in 18 positive cases and in 9 negative cases (ratio 0.67), whereas after exposure up to day/s, in 8 positive papers and in 4 negative results were reported (ratio 0.67).

**Figure 5:**
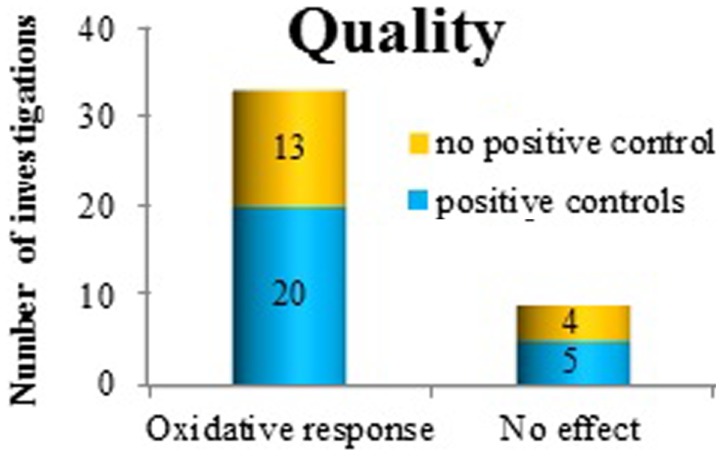
**The use of positive controls in investigating oxidative response as a positive or negative finding after exposure to ELF MF**.

To investigate the *quality* of the studies, where proper sham control conditions, statistical analysis, and other criteria such as blinded protocols were not taken into account (since this would have reduced the number of relevant studies very dramatically), we analyzed how often positive controls were applied as a quality control measure of the study. Of the positive findings, 21 investigations used positive controls. Many of these were investigating co-exposure effects (62%) (Figure 5). Among the negative findings, five papers reported the use of positive controls, all in studies of co-exposure effects.

**Figure 6:**
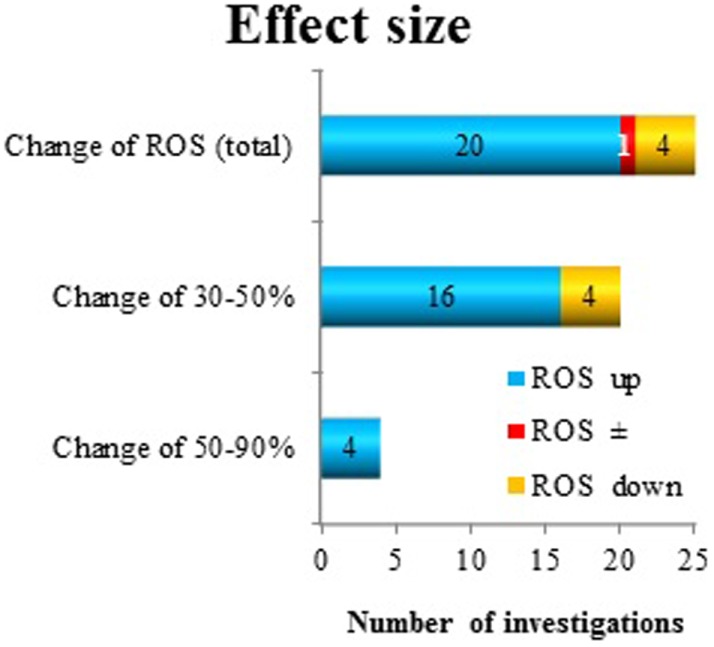
**Reactive oxygen species production as a measure of “effect size” after exposure to ELF MF**. The direction of change (increase: up and decrease: down) has been considered.

A good measure of the “*effect size*” is the most common endpoint among the investigated oxidative responses, namely, superoxide radical anion and/or ROS production. However, the effect size is not amenable for grouping purposes, since it is only one parameter. In 18 publications, superoxide radical anion and/or ROS production were reported positive effects. Of these, 17 showed a change between 30 and 90% and one publication was not reporting the size of the effect. Several investigators analyzed this effect after more than one exposure/co-exposure condition. In total, 20 observations showed an increase (up to 90%) and 4 a decrease (20–40%) and 1 showed no effect due to a certain condition in superoxide radical anion and/or ROS release. However, out of these 25 “datasets,” only four investigations reported a change between 50 and 90%. All other data showed a change in superoxide radical anion and/or ROS production at a level of 30–50% (Figure 6).

In summary, the majority of the reported studies were showing an oxidative response after MF exposure. However, it seems that none of the presented groups are decisive for the induction of oxidative response after exposure to MF.

## Discussion

Health effects research regarding ELF MF has to a large degree been driven by epidemiological findings where certain correlations between long-term exposures and chronic diseases have been found. The most apparent association is the increased risk for childhood leukemia in the presence of elevated domestic exposure levels (1). However, this is an example of a correlation between MF and a disease outcome. There are no experimental findings that can provide a mechanistic explanation for such an outcome. This can be interpreted in two ways: (1) there is no causative association between long-term low-level MF exposure and chronic diseases such as cancer; or (2) the systematic experimental studies that can provide a mechanistic explanation have not been performed.

One of the biological end points that have been frequently investigated is the oxidative status of the biological system. This is due both to that it is reasonable to assume that early responses to external stressors involve changes in oxidative homeostasis, discussed in several recent reviews (57–60) and also that possible lasting effects on the oxidative balance could influence a number of cellular processes in such a way that disease conditions can develop (61–64).

We have published some studies in this field (see Table 1 for pertinent references), and also suggested that an early response to ELF MF is effects on radical homeostasis [e.g., Ref. (13, 14)]. Based on this, we formulated our present hypothesis, viz. that ELF MF exposure consistently triggers oxidative responses in cultured mammalian cells. Taking the complexity of both the exposure situation (with various frequencies, waveforms, modulations, flux densities, presence of other MF, duration, exposure periodization, etc.) and the multitude of biological processes that are more or less relevant endpoints into consideration, it is clear that it is not necessarily easy to test this hypothesis. Therefore, we have decided to use a simple grouping approach to systematically order the available data. Grouping as a tool to make order in, e.g., chemical toxicology situations is well established (65) but has to our knowledge not been used in the field of bioeffects of MF. Nevertheless, we have identified a clear need to use a systematic approach to analyze the published data on the subject of MF exposure and oxidative responses.

What then, has our test of the hypothesis revealed? The outcome of the present analysis, based on relevant available data, is that the hypothesis cannot be rejected. This is due to that the majority of investigated studies showed positive effects, over a broad range of cell types, exposure durations, and flux densities.

What is the support for the statement that the hypothesis is still surviving, but needs to be tested with more precise and distinct approaches? Regarding cell type specific responses, there is no support from the available data to say that specific cell types display more or less sensitivity to the exposure (Figure 1). Rather, positive effects were seen in all the cell type categories that we identified. A caveat is that immune-relevant cells (primary cells as well as cell lines) have been the most frequently used models. This is reasonable, since immune cells employ rapid oxidative responses in their activities. However, since so few studies (i.e., three) of the category “other primary cells” have been studied, further investigations of these cell types would be needed to develop this point further.

When analyzing the responses to various flux densities, several observations are obvious. Most investigations used exposure levels of 1 mT or higher, which almost completely resulted in an oxidative responses, in all investigated cell types. Only 6% of the investigations displayed no effects. Regarding exposures below 1 mT, the outcome is mixed. There are somewhat more positive than negative studies in both lower exposure groups, although the negative findings are well represented as well. Interestingly, there are several positive studies also at or below 0.10 mT. The number of independent studies employing flux densities below 0.10 mT is low, however. Virtually no studies have employed flux densities comparable to environmental levels (single microtesla or lower). Unfortunately, there are very few of the studies that have been performing real dose–response investigations, with several flux density levels. A conclusion regarding a possible threshold effect is also not possible to draw, based on these data. The possibility of a real dose–response pattern does exist, which easily could be addressed in future studies.

Neither the grouping of data based on frequency or modulation (Figure 3) nor exposure duration (Figure 4) showed any support for that very specific conditions are decisive for positive effects. Although most of the studies have been performed at 50/60 Hz, also other frequencies and pulsed MF were represented in both positive and negative findings. A similar pattern is also present when analyzing the importance of exposure duration. Short- (minutes) to long-exposure times (days) were all used in the studies where oxidative responses were noted.

The effect size of the responses in the form of ROS levels were between 30 and 90% change from the control conditions (Figure 6). These changes were mostly increases, although also decreased levels due to exposure were found in a few studies (4 out of 24 studies where ROS responses were seen). Most changes were in the interval 30–50%. This level of change must be considered modest. Various appropriate positive (chemical) controls have been used in some of the studies. These compounds include H_2_O_2_, the phorbol ester TPA, the bacterial endotoxin LPS, and the cytostatic drug cisplatin. The increases in ROS levels due to these chemicals varied from around 40% increase up to several 10-fold increases. The latter large increases correspond to an “oxidative burst” from the cells. This is a recognized characteristic of certain immune cells (57) and has never been seen after MF exposure (13, 14). Whether the small changes caused by MF exposure would have any biological significance is unknown. However, induction of ROS release at lower levels has recently been connected to disease development (61–64).

Regrettably, many studies did not include positive controls (see Figure 5) at all. As many as 40–50% of the studies (both those with and without any effect), lacked this crucial quality control. Obviously, this reduces the usefulness and the credibility of the findings, irrespective of whether they are positive or negative. We also looked for two other study quality markers, i.e., blinded protocols and proper sham exposure conditions. Only a couple of papers were employing both these routines.

Taking the quality criteria together, they weaken the conclusions that can be drawn from these studies. One needs also to keep in mind that the total number of studies that we investigated is relatively few. We furthermore do not know to what extent additional investigations have been unpublished, due to negative findings. Such a publication bias has been documented elsewhere when analyzing EMF biological effects (66). Taking everything into account, our interpretation is that there is evidence supporting that ELF MF cause oxidative responses in mammalian cells, most clearly at higher flux densities (1 mT or higher).

## Conclusion

Available evidence suggests that ELF MF (modulated/unmodulated) has an effect on oxidative status parameters, in both directions. The strongest association between MF exposure and effects occur at or above 1 mT, although effects are noted at or below 0.10 mT. Effects are not dependent on cell type or on exposure duration. Furthermore, the effects are modest in comparison with the responses to positive controls.

## Author Contributions

Both authors have contributed to all parts in the writing process, have approved the final version of the manuscript, and are both fully responsible for the content of the work.

## Conflict of Interest Statement

The authors declare that the research was conducted in the absence of any commercial or financial relationships that could be construed as a potential conflict of interest.

## Footnotes

^1^www.ncbi.nlm.nih.gov

^2^www.emf-portal.de
